# Improving the extraction of complex regulatory events from scientific text by using ontology-based inference

**DOI:** 10.1186/2041-1480-2-S5-S3

**Published:** 2011-10-06

**Authors:** Jung-jae Kim, Dietrich Rebholz-Schuhmann

**Affiliations:** 1 School of Computer Engineering, Nanyang Technological University, Singapore; 2 EMBL-EBI, Wellcome Trust Genome Campus, Hinxton, Cambridge, UK

## Abstract

**Background:**

The extraction of complex events from biomedical text is a challenging task and requires in-depth semantic analysis. Previous approaches associate lexical and syntactic resources with ontologies for the semantic analysis, but fall short in testing the benefits from the use of domain knowledge.

**Results:**

We developed a system that deduces implicit events from explicitly expressed events by using inference rules that encode domain knowledge. We evaluated the system with the inference module on three tasks: First, when tested against a corpus with manually annotated events, the inference module of our system contributes 53.2% of correct extractions, but does not cause any incorrect results. Second, the system overall reproduces 33.1% of the transcription regulatory events contained in RegulonDB (up to 85.0% precision) and the inference module is required for 93.8% of the reproduced events. Third, we applied the system with minimum adaptations to the identification of cell activity regulation events, confirming that the inference improves the performance of the system also on this task.

**Conclusions:**

Our research shows that the inference based on domain knowledge plays a significant role in extracting complex events from text. This approach has great potential in recognizing the complex concepts of such biomedical ontologies as Gene Ontology in the literature.

## Background

The task of extracting events from text, called *event extraction*, is a complex process that requires various semantic resources to decipher the semantic features in the event descriptions. Previous approaches identify and represent the textual semantics of events (e.g. gene regulation, gene-disease relation) by associating lexical and syntactic resources with ontologies [1-5]. We further explore the usage of an ontology for incorporating domain knowledge into an event extraction system.

Events from text that have been hand-curated into relational databases by biologists are actually the products of scientific reasoning supported by the domain knowledge of the biologists. This process of reasoning is based on linguistic evidence of such language patterns as “A regulates B” and “expression of Gene C” which refer to the basic events of regulation and gene expression. These basic events can be combined into an event with the compositional structure “A regulates (the expression of Gene C)”, where the parentheses enclose the embedded event. In this paper, we call such an event consisting of multiple basic events a *complex event* and say that it has a *compositional structure*. We will show that the use of inference based on domain knowledge supports the extraction of complex events from text.

The previous approaches to extracting complex events combine the basic events into compositional structures according to the syntactic structures of source sentences. However, there are two open issues in curating the compositional structures into relational databases. First, the event descriptions in scientific papers are so complicated that it is often required to transform the compositional structures into the structures compatible with the semantic templates of the target databases. Second, an event can be represented across sentence boundaries, even in multiple sentences which are not linked via anaphoric expressions (e.g. ‘it’, ‘the gene’).

Biologists with sufficient domain knowledge have little problem in carrying out the two required tasks of structural transformation and evidence combination. Structural transformation is to find an event that has the same meaning as the original event but with a different structure, while evidence combination is to identify a new event that can be deduced from multiple events. We should encode the domain knowledge into a logical form so that our text mining systems can process the compositional structures of events, which are explicitly expressed in text and can be extracted by language patterns, to deduce the events with alternative structures and those implied by a combination of multiple events. We call the explicitly expressed events *explicit events* and the deduced events *implicit events*.

Several text mining systems have employed inference based on domain knowledge to fill in event templates [6-8]. They can also go beyond sentence boundaries and combine into an event frame the event attributes collected from different sentences. However, they do not use an ontology for representing the inference rules. Moreover, they primarily deal with flat-structured event frames whose participants are physical entities (e.g. protein, residue). To address these issues, we present a novel approach that represents events and domain knowledge with an ontology and combines basic events into a compositional structure where an event participant can be another simpler event.

We utilize Gene Regulation Ontology (GRO), a conceptual model for the domain of gene regulation [9]. The ontology has been designed for representing the compositional semantics of both biomedical text and the referential databases. GRO provides basic concepts and properties of the domain, which are from, and cross-linked to, such biomedical ontologies as Gene Ontology and Sequence Ontology. We use the concepts and properties of GRO to represent the domain knowledge in form of P→Q implications, which we call *inference rules*. We also represent explicit events from text with GRO and apply modus ponens to the inference rules and the explicit events to deduce implicit events.

We implemented a system of event extraction with the proposed inference module and evaluated it on three tasks, reporting that the inference significantly improves the system performance.

## Results

We performed three evaluations to test our system. Each evaluation takes two steps to answer the following two questions, respectively: 1) How well does the system with the inference module extract events from text and 2) how much does the inference module contribute to the event extraction? First, we ran the system on a manually annotated corpus to estimate the performance of the system. Second, we used the system for a real-world task of populating RegulonDB, the referential database of *E. coli* transcription regulatory network, to prove the robustness of the system. The first two evaluations are based on the corpora used for our previously reported experiments [10]. Finally, we applied the system to a related task of extracting regulatory events on cell activities and compared the results with the GOA database [11]. While the first two evaluation tasks focus on *E. coli*, a prokaryotic model organism, the last task deals with human genes and cells.

Table 1 shows the event templates for the evaluations. The first two evaluations are to extract instances of the first three event templates in the table, while the last evaluation is to extract instances of the two last event templates. Our system deals with four properties of events: 1) agents which bind to gene regulatory regions or control gene expression and cell activities; 2) patients which are regulated by the agents; 3) polarity, which tells whether the agent regulates the patient positively or negatively; and 4) physical contact, which indicates whether the agent regulates the patient directly by binding or indirectly through other agents. Since the three evaluations only consider the agents and patients, the event templates in Table 1 include only the two properties.

**Table 1:** Semantic templates for target events

Semantic template	Gene Ontology concept
<RegulationOfGeneExpression hasAgent=?Protein hasPatient=<GeneExpression hasPatient=?Gene>>	Regulation of gene expression (GO:0010468)
<RegulationOfTranscription hasAgent=?Protein hasPatient=<Transcription hasPatient=?Gene>>	Regulation of transcription (GO:0045449)
<BindingOfTFToTFBindingSiteOfDNA hasAgent=?TranscriptionFactor hasPatient=<RegulatoryDNARegion hasPatient=?Gene>>	Transcription factor binding (GO:0008134)
<RegulatoryProcess hasAgent=?MolecularEntity hasPatient=<CellGrowth hasAgent=?Cell>>	Regulation of cell growth (GO:0001558)
<RegulatoryProcess hasAgent=?MolecularEntity hasPatient=<CellDeath hasAgent=?Cell>>	Regulation of cell killing (GO:0031341)

### Evaluation against event annotation

We evaluated our system first against a manually annotated corpus. The corpus consists of 209 MEDLINE abstracts that contain at least one *E. coli* transcription factor (TF) name. Two curators have annotated *E. coli* gene regulatory events on the corpus and have agreed on the final release of the annotated corpus which is available at http://www.ebi.ac.uk/~kim/eventannotation/ (see [10] for details, including inter-annotator agreement).

We randomly divided the corpus into two sets: One for system development (i.e. training corpus) and the other for system evaluation (i.e. test corpus). The training corpus, consisting of 109 abstracts, has 250 events annotated, while the test corpus, consisting of 100 abstracts, has 375 events annotated. We manually constructed language patterns and inference rules, based on the training corpus and a review paper (see the Methods section for details).

The system successfully extracted 79 events from the test corpus (21.1% recall) and incorrectly produced 15 events (84.0% precision). We consider an extracted event as correct if its two participants and their roles (i.e. agent, patient) are correctly identified, following the evaluation criteria of the previous approaches [3,12]. Among the 79 events, the system has correctly identified polarity of 46 events (58.2% precision) and physical contact of 51 events (64.6% precision), while these two features are not considered for estimating the system performance, following the evaluation criteria of the previous approaches [3,12]. To understand the contribution of the inference on the system, we have run the system without the inference module. It then extracts only 37 out of the successfully extracted 79 events, which indicates that the inference contributes on 53.2% of the correct results. In addition, the inference was involved in the extraction of only three out of the 15 incorrectly extracted events. This result supports our claim that logical inference can effectively deduce implicit textual semantics from explicit textual semantics. We have further focused on the events whose agents are TFs for the purpose of comparing our system with [3,12]. The test corpus has 305 events with TFs as agents. The system has successfully extracted 66 events among them (21.6% recall) and incorrectly produced 6 events (91.7% precision). This performance is slightly better than that of [3] (90% precision, ~20% recall) and of [12] (84% precision).

We analyzed the errors of the system as follows: The false positives, in total 15 errors, are mainly due to the inappropriate application of the loose pattern matching method (7 errors) (see the Methods section for details). The other causes include parse errors (2), the neglect of negation (1), and an error in conversion from predicate argument structure to dependency structure (1). These results of error analysis indicate that the three incorrect events, which were extracted by the system with the inference module, are actually due to the incorrect outputs of the prior modules (e.g. pattern matching) passed to the inference module. In short, the inference module caused no incorrect results.

We also analyzed the false negatives. We found that 29.7% of the missing events (88/296) are due to the deficiency of the gene name dictionary and that 30.0% (68/296) are due to the lack of anaphora resolution. The rest of the missing events (40.3%) are thus dependent upon pattern matching and inference. It is hard to distinguish errors by pattern matching from those by the inference, because the inference module takes into consideration all semantics from an entire document (i.e. MEDLINE abstract) for the evidence combination. Therefore, the inference together with the pattern matching affects at most 40% of the false negatives.

### Evaluation against RegulonDB

We tested the system against the real-world task of populating RegulonDB with *E. coli* transcriptional regulatory events from the literature. We used four corpora that are relevant to *E. coli* transcription regulation [10]: 1) the regulon.abstract corpus with 2,704 MEDLINE abstracts which are references of RegulonDB, 2) the regulon.fulltext corpus with the fulltexts of 436 references in RegulonDB, 3) the ecoli-tf.abstract corpus with 4,347 MEDLINE abstracts that contain at least one *E. coli* TF name, and 4) the ecoli-tf.fulltext with the fulltexts of 1,812 papers among those in the ecoli-tf.abstract.

We have measured the performance of the system for this evaluation task as follows: The precision is measured as the percentage of events found in RegulonDB among the unique events extracted by the system, while the recall is the percentage of the successfully extracted events among those curated in RegulonDB. The version of RegulonDB used for the evaluation is 6.2, containing 4,579 *E. coli* genes, 169 TFs, and 3,590 unique gene regulation events. This evaluation only considers events with TFs as agents because of the purpose of populating RegulonDB. The overall performance is as follows: F-score 0.44, precision 66.6%, and recall 33.1%. Table 2 shows the evaluation results over each test corpus, where the performance of the system without the inference is displayed within pairs of parentheses.

**Table 2:** Evaluation against RegulonDB

Corpus	Recall	Precision	F-score
ecoli-tf.abstract	22.4% (0.3%)	77.2% (50.0%)	0.35 (0.01)
ecoli-tf.fulltext	24.0% (1.5%)	67.1% (76.1%)	0.35 (0.03)
regulon.abstract	17.1% (0.1%)	85.0% (80.0%)	0.28 (0.00)
regulon.fulltext	14.1% (1.2%)	74.0% (91.7%)	0.24 (0.02)
Total	33.1% (2.1%)	66.6% (79.6%)	0.44 (0.04)

Additionally, we analyzed the effect of event types. The precision for the events of the type “regulation of transcription” is 85%, higher than that of [12] (77% precision), while the overall precision (67%) is predictably lower than that since the system of [12] is developed specifically for extracting regulatory events on gene transcription. We included the events of the other two types, which are hypernyms of “regulation of transcription”, into the result set for the evaluation, because of the low recall for the events of “regulation of transcription” (5%). The overall recall (33%) is still lower than that of [12] (45% recall) because of the small size of the regulon.fulltext corpus (436 fulltexts). Note that [12] extracted 42% of RegulonDB events from 2,475 fulltexts of RegulonDB references. We plan to analyze a larger number of fulltexts in the future.

It is remarkable that the inference is inevitable for extracting 93.8% of the RegulonDB events that are extracted by our system from the corpora. In contrast, the inference module is involved in the extraction of only 3.2% of the false negative events. The percentage 93.8% is much higher than 53.2% of the first evaluation. The difference may be due to the fact that this second evaluation only counts unique events, while the first evaluation against the event annotations counts all extracted event instances. If so, these results may indicate that only a small amount of well-known events are frequently mentioned in papers in concise language forms, thus extracted by language patterns even without the help of inference, and that the rest of the events are expressed in papers with the detailed procedures of experiments which led to the discovery of the events.

### Adaptation for regulation of cell activities

Rule-based systems are criticized for being too specific to the domains for which they have been developed, so much so that they cannot be straightforwardly adapted for other domains. To prove the adaptability of our system, we have applied it to a related topic: Regulation of cell activities.

The goal of this new task is to populate the GOA [11], concerning two Gene Ontology (GO) concepts: Regulation of cell growth (GO:0001558) (shortly, RCG) and regulation of cell death (GO:0031341) (shortly, RCD). GOA is a database which provides GO annotations to proteins. In short, the task is to identify the proteins that can be annotated with the two GO concepts. The semantic templates of the two event types are defined in Table 1.

The adaptation included only the following work: We manually collected keywords of the concepts ‘growth’ and ‘death’ from WordNet and constructed 40 patterns for the keywords by using MedEvi [13]. As candidate agents, we collected human gene/protein names from UniProt. We also collected cell type names from MeSH. These are newly built resources that were not required for the first two evaluation tasks. Existing language patterns and inference rules, for example for the concept ‘regulation’, were reused. We have not used any training corpus to further adjust the system to the new task.

We constructed a test corpus consisting of 13,136 abstracts by querying PubMed with two MeSH terms “Cell Death” and “Cell Enlargement”. The system with the inference module extracted 244 unique UniProt proteins associated with RCG events and 266 unique proteins associated with RCD events from the corpus. This evaluation also uses the two measures: Precision, the percentage of unique proteins found in GOA among the extracted proteins, and recall, the percentage of extracted proteins among the protein records in GOA. GOA contains 16 proteins among the 244 proteins of RCG events (6.6% precision) and 100 proteins among the 266 proteins of RCD events (37.6% precision). Currently (2010 July), the GOA has 155 proteins associated with RCG (10.3% recall) and 908 proteins associated with RCD (11.0% recall). These results show that our system can be applied to a related task with minimal adaptations.

We also tested the system without the inference module against the cell corpus. It identifies 193 proteins associated with RCG events and 198 proteins associated with RCD events. GOA contains 13 proteins among the 193 proteins of ROG events (6.7% precision) and 78 proteins among the 198 proteins of RCD events (39.4% precision). The precision almost does not change even after running without the inference module, while the recall drops about 20% without the inference module. This finding is similar to what we found from the results of the second evaluation such that the precision is independent from the inference, while the recall drops significantly without the inference module. But the relatively smaller drop of recall for the new task may indicate that the inference rules developed for the first two evaluations have less effects on the third evaluation than the other two evaluations.

We have manually inspected 20 out of the proteins that are extracted by our system but not found in GOA, for each event type. Among the 20 ‘false positive’ proteins of the RCD concepts, we found evidence that can support the association of 15 proteins with RCD concepts (75%). This means that the real precision can go up to 80% and more importantly that we can identify new protein instances of GO concepts by using our system. Among the 20 ‘false positive’ proteins of the RCG concepts, we located evidence only for 8 proteins (40%). After careful inspection, we realized that the precision of the RCG-related proteins is much lower than that of the RCD-related proteins because the language patterns for RCG events, which we collected from WordNet, are not specific to cell size growth, but may also refer to cell proliferation and development which should be linked to the other GO concepts “cell proliferation” (GO:0008283) and “cell development” (GO:0048468). The lack of training corpus led to this problem, and so we plan to extend the experiment to other GO concepts, establishing training corpora for the concept identification in text.

## Discussion

As explained in the Introduction, the inference rules we introduce in this paper are to deduce implicit events from explicit events. Note that unless the explicit events contain enough evidence to an implicit event, we cannot make logical deduction of the implicit event. In other words, the implicit events are alternative representations of the extracted information, where the implicit events do not convey ‘new’ information compared to the explicit events. The performance comparison between the system with the inference and that without the inference is, in a sense, to see which representations better fit for the target templates, where the inference rules are designed to produce results that better match the target templates. Previous systems often embed linguitic and domain knowledge required for event extraction together into hand-crafted rules or machine learning models, thus biased to target templates. In contrast, our approach of separating the inference rules from the linguistic resources helps us construct language patterns without respect to target templates [5]. Considering the compositional aspect of events, it leads us to the development of phrase-level patterns, which are close to lexical semantics, rather than sentence-level patterns [14]. In addition, we may associate the lexical patterns with the well-defined semantic types of an ontology and focus on the semantic types that are related to a given application task, not worrying about the side-effect of domain-specific patterns. This makes the patterns highly reusable, as shown in the third test case.

## Conclusions

We proposed a novel approach to event extraction, using an ontology to represent the semantics of lexical, syntactic, and pragmatic resources. We focused on extracting regulatory events on gene expression and cell activities, which are very important to molecular biology and disease studies. Our system shows the full complexity in the identification of such complex events from the literature and may guide the ontology development to innovative ways of integrating various knowledge resources.

## Methods

Our system first recognizes mentions of individual GRO instances in text, which can be the event components. It then combines them into compositional structures of explicit events by using language patterns. The system performs inference based on domain knowledge to deduce implicit events from the explicit events. It finally extracts the events that match pre-defined event templates. Both explicit and implicit events may fit for the database event templates.

**Figure 1:**
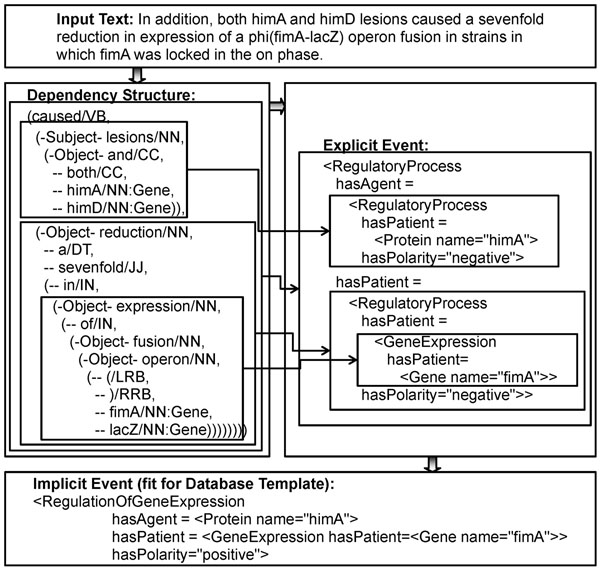
**Example 1 of event extraction**. The figure depicts the three types of structures from the input text: Dependency structure, explicit events, and implicit events. An arrow between the syntactic and semantic structures indicates a correspondence link between two structures for a phrase. The explicit event is combinatorially composed from phrasal structures to sentential structures by using the patterns in Table 3. The implicit event is deduced from the explicit events by using the inference rules 1 to 3 in Table 4.

**Figure 2:**
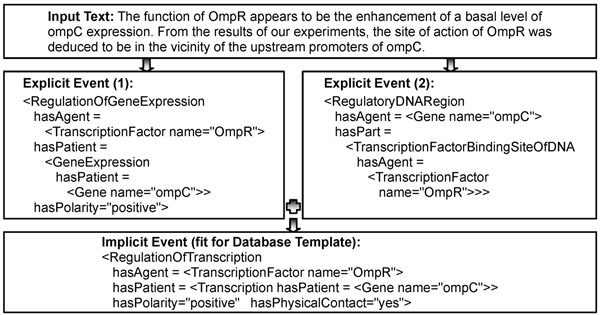
**Example 2 of event extraction**. The figure shows that the explicit events of the two sentences are combined to deduce the implicit event. Rule 4 in Table 4 is used for the deduction.

**Figure 3:**
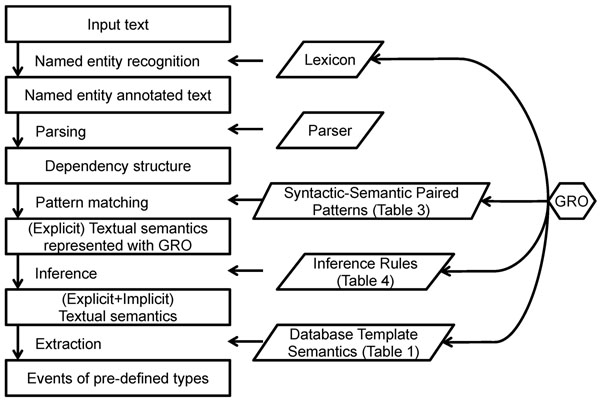
**System workflow**. The boxes represent input, output, and intermediate results of the system, while the labels in-between indicate the modules of the system. The parallelograms indicate the resources utilized by the system modules. The hexagon refers to the GRO which is used to represent the semantic information in the resources.

Figures 1 and 2 show the examples of the extracted events. Figure 1 depicts the three types of structures from the input text: Dependency structure, explicit event, and implicit event. An arrow between the syntactic and semantic structures indicates a correspondence link between two structures for a phrase. The explicit event is composed from phrasal structures to sentential structures by using the patterns in Table 3. The implicit event is deduced from the explicit events by using the inference rules 1 to 3 in Table 4. TFBS stands for TranscriptionFactorBindingSiteOfDNA. Figure 2 shows that the explicit events of the two sentences are combined to deduce the implicit event. Rule 4 in Table 4 is used for the deduction. The overall workflow of the system is depicted in Figure 3.

**Table 3:** Example patterns

No.	Syntactic pattern	Semantic pattern
1	(expression_Noun (of Prep Object:Gene))	<GeneExpression hasPatient=Gene>
2	(reduction_Noun (in Prep Object:Patient))	<RegulatoryProcess hasPatient=Patient hasPolarity=“negative”>
3	(lesion_Noun Object:Patient)	< RegulatoryProcess hasPatient=Patient hasPolarity=“negative”>
4	(cause_Verb Subject:Agent Object:Patient)	<RegulatoryProcess hasAgent=Agent hasPatient=Patient>

**Table 4:** Example inference rules

No.	Condition(s) ⇒ Conclusion
1	<RegulatoryProcess hasPolarity=Polarity2 hasAgent=<RegulatoryProcess hasPatient=Patient hasPolarity=Polarity1>> ⇒ <RegulatoryProcess hasAgent=Patient hasPolarity=polarity_sum(Polarity1,Polarity2)
2	<RegulatoryProcess hasPolarity=Polarity2 hasPatient=<RegulatoryProcess hasPatient=Patient hasPolarity=Polarity1>> ⇒ <RegulatoryProcess hasPatient=Patient hasPolarity=polarity_sum(Polarity1,Polarity2)
3	<RegulatoryProcess hasPatient=GeneExpression> ⇒ <RegulationOfGeneExpression hasPatient=GeneExpression>
4	<RegulationOfGeneExpression hasAgent=TranscriptionFactor hasPatient=<GeneExpression hasPatient=Gene>> + <RegulatoryDNARegion hasAgent=Gene hasPart=<TFBS hasAgent=TranscriptionFactor>> ⇒ <RegulationOfTranscription hasAgent=TranscriptionFactor hasPatient = <Transcription hasPatient=Gene> hasPhysicalContact=“yes”>

### Named entity recognition

We have adopted a dictionary-based approach for named entity recognition. The dictionary contains 15,881 gene/protein and operon names of *E. coli*, including the names of 169 *E. coli* TF names, collected from RegulonDB and SwissProt. The recognized names are grounded with UniProt identifiers and labeled with relevant GRO concepts among the followings: Gene, Protein, Operon, and TranscriptionFactor.

### Parsing

We have utilized Enju, the HPSG parser [15], for syntactic analysis of sentences. While the Enju parser produces predicate-argument structures, we have developed a module to convert them into dependency structures and selectively merged the predicate-argument structure into the dependency structure. We have identified the dependency structure for the loose matching of language patterns explained below.

### Pattern matching

To identify the explicit events from sentences, the system utilizes syntactic-semantic paired patterns, matching the syntactic patterns to the dependency structures and combining the semantic patterns into a semantic structure.

Each pattern is a pair of a syntactic pattern and a semantic pattern. Syntactic patterns comply with dependency structures. The leftmost item within a pair of parentheses (e.g. cause Verb, lesion Noun) is the head of the other items within the parentheses (e.g. Subject:Agent, Object:Patient). A dependent item may be surrounded by another pair of parentheses, which forms an embedded structure (e.g. Pattern 1, Pattern 2). The lexical items in the syntactic patterns are labeled with part-of-speech (POS) tags (e.g. Verb, Noun, Prep), and should be matched to words with the same POS tags. The dependent items have syntactic constraints that indicate their roles with respect to their head items (e.g. Subject, Object), and should be matched to those with the syntactic roles. The dependent items may have semantic variables (e.g. Agent, Patient, Gene), which indicate the semantics of the dependent items. If the semantic variable of a dependent item is a concept of GRO (e.g. Gene), the variable should match a semantic category that is identical to, or a sub type of, the specified concept.

The semantic pattern expresses the semantics of its corresponding syntactic pattern. The semantic pattern is represented with GRO concepts (e.g. RegulatoryProcess, GeneExpression) and properties (e.g. hasAgent, hasPatient).

The system tries to match the syntactic patterns to the dependency structures of sentences in a bottom-up way. For example, it matches from Pattern 1 to Pattern 4 in Table 3 to the dependency structure of the example (1) depicted in Figure 1. In the process, it considers the syntactic and semantic constraints of the syntactic patterns. For instance, the item ‘cause’ of the fourth pattern in Table 3 should match the verb ‘cause’ that has both a subject and an object.

Once a syntactic pattern is successfully matched to a node of dependency structure, its corresponding semantic pattern is assigned to the node as one of its semantics. If the syntactic pattern has dependent items with semantic variables (e.g. Subject:Agent, Object:Patient), the variables (e.g. Agent, Patient) are replaced with the semantics of the children of the node that have been matched to the dependent items. In this way, the semantics of multiple phrases is combined into sentential semantics. In Figure 1, the small boxes with dashed lines show the semantics assigned to the internal nodes of the example (1), which are later combined into the textual sentential semantics.

Note that the node ‘lesions’ is assigned two pieces of semantics for the two gene names that are the children of the node (i.e. himA, himD). The explicit textual semantics of Figure 1 is one of the two, while the other is a duplicate of Sem1 except that the gene name ‘himA’ is replaced with ‘himD’.

One important feature of the pattern matching is that we loosely match the syntactic patterns to the dependency structures. For instance, the gene name ‘fimA’ is not a direct child of the preposition ‘of’, but is matched to the item Object:Gene of the first pattern in Table 4. We have decided to match a dependent item not only to a direct child of the node matched to the head item, but also to any descendant of the node. The feature is based on two reasons: First, it is practically impossible to construct all potential patterns for the event extraction, though a reasonably large number of patterns for gene regulation have been accumulated; and second, the lexical entries not matched to any of the patterns for gene regulation (e.g. ‘sevenfold’, ‘operon’, ‘fusion’) might not affect the extraction of the events.

This loose matching still works under the following strict conditions: 1) An item with a syntactic role (e.g. Subject) can be matched to one of descendants under the sub-tree with the syntactic role; 2) once an item is matched to a node, it is not further matched to the node’s descendants; and 3) it does not jump over clausal boundaries (e.g. ‘which’) and several exceptional words (e.g. ‘except’).

### Inference

The inference step is to transduce explicit textual semantics (or events) into implicit semantics (or events). It deduces a new specific event instance, if possible, by combining any two or more general events. The inference module takes as input the explicit events from a text (i.e. a MEDLINE abstract, a fulltext) identified by the previous module of pattern matching. It applies to the explicit events the inference rules that reflect common sense knowledge and domain knowledge, as exemplified in Table 4. An inference rule has the propositional logic form of *P* → *Q*, where P is a set of conditions and Q is the conclusion. It works with the modus ponens rule (i.e. *P*, *P*→*Q* ⊦ *Q*)*.* That is, if all the conditions P of a rule match some of the identified events from a text, the conclusion Q is instantiated and then added as an additional event of the text. As the input events are represented with GRO, the inference rules and their resultant events are also represented with GRO.

We have constructed 28 inference rules for dealing with the compositional structures of gene regulation events (e.g. Rules 1, 2) and for deducing biological events from the combination of linguistic events (e.g. Rules 3, 4) by consulting the training corpus and the review paper [16] (see Table 4).

For example, Rules 1 and 2 flatten, if possible, the compositional structure of event descriptions. The explicit events in Figure 1 has a cascaded structure with four basic event instances (i.e. three RegulatoryProcess, one GeneExpression) and is transformed by Rules 1 and 2 to fit for the database template that has only two event instances (i.e. RegulationOfGeneExpression, GeneExpression). Rule 3 deduces the specific event type RegulationOfGeneExpression from a general type of event (i.e. RegulatoryProcess). Rule 4 reflects the domain knowledge that if a transcription factor both binds to the regulatory region of a gene and regulates the gene’s expression level, it is the transcriptional regulator of the gene. Note that the two conditions of Rule 4 can be matched to events from any sentences; in other words, Rule 4 can merge multiple evidence from different sentences into a fact. The function polarity_sum works exactly like NXOR (Not Exclusive OR) operation in Boolean logic. The rules are repeatedly applied over the explicit events from a given text until no additional event is generated.

We have implemented a program that converts the inference rules into Prolog programming codes and a Prolog application that executes the rules over input events. We could not use the OWL-DL reasoners (e.g. Pellet) because of the DL-safe restriction of the reasoners. DL-safe restriction assumes that all instances of rules, both in conditions and in conclusions, should be available at the knowledge base [17]. Unfortunately, however, the rules for the event extraction generate new instances of events and event attributes in the conclusions. Nonetheless, we can still utilize the reasoners to validate the ontology populated with the extracted events.

### Extraction

The system finally selects the events that match given semantic templates among those resulted from either pattern matching or inference. Table 1 shows the event templates. The variables are marked with ‘?’ and are matched to the instances of the concepts referred to by the variables. For example, the variable “?Protein” can be matched to a protein name. Non-variable concepts and properties are used as semantic restriction on the events to extracted. For example, the last template in Table 1 can be matched to an instance of NegativeRegulation, which a child of RegulatoryProcess. In addition, the patient of the instance should an instance of CellDeath and the agent can be a gene, where Gene is a descendant of MolecularEntity.

## Competing interests

The authors declare that they have no competing interests.

## Authors contributions

JJK conceived the study, designed and implemented the system, carried out the evaluations and drafted the manuscript. DRS motivated and coordinated the study and revised the manuscript.

## References

[B1] Daraselia N Yuryev A Egorov S Novichkova S Nikitin A Mazo I Extracting human protein interactions from MEDLINE using a full-sentence parser Bioinformatics 2004 20 5 604 611 10.1093/bioinformatics/btg452 15033866

[B2] Cimiano P Reyle U Saric J Ontology-based discourse analysis for information extraction Data & Knowledge Engineering 2005 55 59 83 10.1016/j.datak.2004.11.009

[B3] Saric J Jensen LJ Rojas I Large-scale extraction of gene regulation for model organisms in an ontological context Silico Biology 2005 5 21 32 15972005

[B4] Hunter L Lu Z Firby J Baumgartner WA Johnson HL Ogren PV Cohen KB OpenDMAP: an open source, ontology-driven concept analysis engine, with applications to capturing knowledge regarding protein transport, protein interactions and cell-type-specific gene expression BMC Bioinformatics 2008 9 78 10.1186/1471-2105-9-78 18237434 PMC2275248

[B5] Kim JD Ohta T Pyysalo S Kano Y Tsujii J Overview of BioNLP’09 shared task on event extraction Proceedings of the Workshop on BioNLP: Shared Task 2009 1 9

[B6] Gaizauskas R Demetriou G Artymiuk PJ Willett P Protein structures and information extraction from biological texts: The PASTA system Bioinformatics 2003 19 135 143 10.1093/bioinformatics/19.1.135 12499303

[B7] Narayanaswamy M Ravikumar KE Vijay-Shanker K Beyond the clause: extraction of phosphorylation information from medline abstracts Bioinformatics 2005 21 Suppl. 1 i319 i327 15961474 10.1093/bioinformatics/bti1011

[B8] Culotta A McCallum A Betz J Integrating probabilistic extraction models and data mining to discover relations and patterns in text Proceedings of Human Language Technology Conference of the North American Chapter of the Association of Computational Linguistics 2006 296 303

[B9] Beisswanger E Lee V Kim JJ Rebholz-Schuhmann D Splendiani A Dameron O Schulz S Hahn U Gene Regulation Ontology (GRO): Design Principles and Use Cases Studies in Health Technology and Informatics 2008 136 9 14 18487700

[B10] Hahn U Tomanek K Buyko E Kim JJ Rebholz-Schuhmann D How Feasible and Robust is the Automatic Extraction of Gene Regulation Events? A Cross-Method Evaluation under Lab and Real-Life Conditions Proceedings of BioNLP 2009 2009 37 45

[B11] Barrell D Dimmer E Huntley RP Binns D O’Donovan C Apweiler R The GOA database in 2009 - an integrated Gene Ontology Annotation resource Nucleic Acids Research 2009 37 D396 D403 10.1093/nar/gkn803 18957448 PMC2686469

[B12] Rodríguez-Penagos C Salgado H Martínez-Flores I Collado-Vides J Automatic reconstruction of a bacterial regulatory network using Natural Language Processing BMC Bioinformatics 2007 8 293 10.1186/1471-2105-8-293 17683642 PMC1964768

[B13] Kim JJ Pezik P Rebholz-Schuhmann D MedEvi: Retrieving textual evidence of relations between biomedical concepts from Medline Bioinformatics 2008 24 11 1410 1412 10.1093/bioinformatics/btn117 18400773 PMC2387223

[B14] Kim JJ Chae YS Choi KS Phrase-Pattern-based Korean to English Machine Translation using Two Level Translation Pattern Selection Proceedings of 38th Association for Computational Lingusitics (ACL) 2000 31 36

[B15] Sagae K Miyao Y Tsujii J HPSG parsing with shallow dependency constraints Proceedings of the 45th Annual Meeting of the Association of Computational Linguistics Prague, Czech Republic 2007 624 631

[B16] Browning DF Busby SJW The regulation of bacterial transcription initiation Nature Reviews Microbiology 2004 2 57 65 10.1038/nrmicro787 15035009

[B17] Motik B Sattler U Studer R Query answering for OWL-DL with rules Web Semantics: Science, Services and Agents on the World Wide Web 2005 3 41 60

